# Edible Cannabis Product Information Captured Within Patient Poison Center Records

**DOI:** 10.1192/j.eurpsy.2026.10875

**Published:** 2026-07-31

**Authors:** G. Fulkerson, B. J. Holstege, R. Farah, A. Zmuda, C. P. Holstege

**Affiliations:** 1 Emergency Medicine, University of Virginia, Charlottesville; 2 Psychiatry, Old Dominion University, Norfolk, United States

## Abstract

**Introduction:**

United States poison centers are encountering increasing numbers of calls pertaining to edible cannabis exposed patients following state decriminalization. The emergence of these edible products has led to numerous emergency department visits in both adults and children following exposure. The capturing of specific data associated with the product type is imperative as state government regulators attempt to control cannabis products emerging on the market as a growing public health issue.

**Objectives:**

This study was performed to determine if the exact products involved with edible cannabis exposure were being captured in the electronic medicial records and to delineate those specific edible products.

**Methods:**

This retrospective patient records review surveys a sampling of Toxicall electronic medical records associated with a poison center from 1 January 2024 to 31 December 2024. Edible cannabis product human exposure records from calls to a single poison center (n=157) were manually evaluated for demographics, therapy outcome, and product information. Documented data were analyzed, focusing on 1) missing product information, and 2) differences in pediatrics aged 0-9, aged 10-20 years, and legal-aged consumer (≥21 years) exposures & product information provided.

**Results:**

Demographics, therapy outcome, and amount of product ingested were always requested per documentation. Minimal product vendor (n=6), cannabis use history (n=11), or labeled serving size (n=12) information was requested. Twelve cannabinoids and 16 POISINDEX codes were recorded; unknown cannabinoids characterize 23% of cases.

Of the products described (n=119), most (n=95, 80%) were gummies. The next most common formulations were non-gummy lookalike candy (products with significant naming / font / color similarities to a branded non-cannabinoid product, n=8), chocolate candy (n=5), and brownies (n=3). Product type was most described in pediatrics aged 0-9 (84.3%), followed by legal-aged consumers (71.2%) and ages 10-20 (69.2%). Six of the eight lookalike products were reported in pediatric cases. One-third of cases (n=52) recorded specific product names. In 73% of cases where a product name was requested, the product name was provided. Information exchanged in exposures involving adult patients were slightly more likely to include a product name in comparison to exposures involving pediatric patients (25.7% versus 22.4%).

**Conclusions:**

When specifically requested, the majority of callers provided the ingested edible cannabis product name, but this information was not requested in two-thirds of cases. Gummy formulations were particularly common and young pediatric patients are disproportionately reflected in product exposures. Improved poison center data collection pertaining to specific cannabis products would enable better tracking of adverse outcomes with those products as a critical part of public health surveillance.

**Disclosure of Interest:**

None Declared

